# Who Engaged in Home-Based Arts Activities During the COVID-19 Pandemic? A Cross-Sectional Analysis of Data From 4,731 Adults in the United States

**DOI:** 10.1177/15248399221119806

**Published:** 2022-09-01

**Authors:** Jessica K. Bone, Hei Wan Mak, Jill K. Sonke, Meg E. Fluharty, Jenny B. Lee, Anthony J. Kolenic, Heidi Radunovich, Randy Cohen, Daisy Fancourt

**Affiliations:** 1 University College London, London, UK; 2 University of Florida, Gainesville, FL, USA; 3 Americans for the Arts, Washington, DC, USA

**Keywords:** arts engagement, cultural engagement, lockdown, stay-at-home orders, social gradient, health, wellbeing, United States

## Abstract

Arts engagement is a health-related behavior that may be influenced by social inequalities. While the COVID-19 pandemic provided new opportunities for some people to engage in the arts, it might have created barriers for others. We aimed to examine whether there was social patterning in home-based arts engagement during the pandemic in the United States, and whether predictors of engagement differed according to the type of arts activity. We included 4,731 adults who participated in the United States COVID-19 Social Study between April and July 2020. Three types of home-based arts engagement were considered: reading for pleasure, arts or crafts activities, and digital arts activities. Using logistic regression models, we tested cross-sectional associations between a broad range of demographic, socioeconomic, psychosocial, and health-related factors as well as adverse events and worries during lockdown and each type of arts engagement. The factors most strongly associated with all three types of arts engagement were social support, social network size, age, race/ethnicity, keyworker status, and experiencing physical or psychological abuse during the pandemic. However, most socioeconomic and health-related factors were not associated with arts engagement, including household income and mental and physical health problems. Overall, our findings indicate that the social gradient in arts engagement was reduced in the first 4 months of the COVID-19 pandemic in the United States. Given the health benefits of arts engagement, the potential diversification of arts audiences during the pandemic is promising for both population-level health and wellbeing and the future of the arts and cultural sector.

In 2019, the World Health Organization identified more than 3000 studies showing the beneficial impact of arts engagement on health and wellbeing (Fancourt & Finn, 2019). The arts may support the prevention and promotion of health by influencing the social determinants of health, encouraging health-promoting behaviors, preventing ill health, and supporting caregiving, as well as supporting the management and treatment of existing health problems, such as mental illness, acute conditions, neurodevelopmental and neurological disorders, noncommunicable diseases, and end-of-life care (Fancourt & Finn, 2019). The INNATE Framework was recently developed to describe the active ingredients of arts activities that may support health, identifying a wide range of important factors, from providing opportunities for creative expression and aesthetic pleasure to cognitive stimulation to social interaction (Warran et al., 2022). These ingredients activate causal mechanisms that lead to effects on health and wellbeing. According to the Multi-level Leisure Mechanisms Framework, there are over 600 mechanisms through which arts engagement might influence health and wellbeing, including psychological processes (e.g., improving self-esteem and mood), biological processes (e.g., reducing levels of stress hormones and inflammation), social processes (e.g., reducing loneliness and improving communication), and behavioral processes (e.g., enhancing agency and motivation to engage in other health behaviors; Fancourt et al., 2021).

These benefits of arts engagement appear to have endured throughout the current coronavirus (COVID-19) pandemic. Spending more time on hobbies such as painting, writing, and other creative activities was associated with reductions in symptoms of depression and anxiety and improvements in life satisfaction during the first lockdown in the United Kingdom (Bu et al., 2021). People have reported using creative activities and consuming media to cope during lockdown, and arts activities helped frontline health and social care professionals to cope with challenges of the pandemic, supporting their mental health (Aughterson et al., 2021; Wright et al., 2021). Given the number of people experiencing distress, depression, anxiety, and loneliness during the COVID-19 pandemic (Bu et al., 2020; Holman et al., 2020), arts engagement is an important health behavior for improving the mental health and wellbeing of the population during the pandemic.

Despite unprecedented challenges for the arts and cultural sector during the COVID-19 pandemic, including the closure of community venues (Guibert & Hyde, 2021), home-based arts engagement may have increased when people were directed to stay at home. There were large increases in online sales of arts and crafts products at the start of the pandemic (Choi et al., 2020). In a large survey of 19,000 adults in the United Kingdom, nearly a quarter reported increases in their arts engagement during lockdown (Mak et al., 2021). These increases in arts engagement were likely due to a range of factors, including more leisure time; using the arts to cope with boredom, stress, or health problems; using the arts to connect with others socially; and the increased availability and promotion of virtual resources, including online groups (e.g., choirs and book groups), streamed performances (e.g., plays and concerts), and digital arts activities (e.g., virtual museum tours). However, while the pandemic provided new opportunities for some people to engage in the arts, it might have created barriers for others. Prepandemic surveys have found that arts engagement differs according to a range of demographic and socioeconomic factors, including gender, education, income, perceived social class, residential area, and childhood socioeconomic status (Bone et al., 2021; Mak et al., 2020, 2021; Stallings & Mauldin, 2016). Generally, people are more likely to engage in the arts if they have a higher socioeconomic position. These factors mirror those that contribute to the inequalities in access to health care and health and social outcomes. But, given that more people may have engaged in the arts during the pandemic, it is unclear whether this social gradient in arts engagement was increased, maintained, or even reduced at the start of the pandemic.

In the United Kingdom, one large study found that older adults, those with less education, and males had lower levels of arts engagement both before and during the pandemic (Mak et al., 2021). Despite this, those who had traditionally been excluded from the arts, such as people of ethnic minorities and those with health conditions, had similar levels of home-based arts engagement compared to others in the pandemic (Mak et al., 2021). However, a parallel study did not find evidence for changes in arts audiences in the United Kingdom, suggesting that preexisting inequalities in arts engagement were maintained (Feder et al., 2022). In addition to these findings being inconsistent, it is unclear how they will generalize to the United States. To our knowledge, no research has yet explored the predictors of arts engagement in the United States during the pandemic. The different social, cultural, and demographic context of the United States, coupled with inconsistent, decentralized COVID-19 restrictions across states, could mean that the predictors and patterns of arts engagement during the pandemic differed to other countries. In addition, different job subsidy structures could have had distinct impacts on arts workers in the United States and United Kingdom, affecting the provision of the arts during the pandemic.

Therefore, in this study, we examined whether there was social patterning in home-based arts engagement during the pandemic in the United States, and whether predictors of engagement differed according to the type of arts activity. We analyzed data from a large sample recruited during the first four months of stay-at-home directives. Three types of arts engagement were considered: reading for pleasure, arts or crafts activities, and digital arts activities. These forms of engagement are health behaviors that have been shown to benefit mental and physical health and wellbeing, both before and during the pandemic (Bu et al., 2021; Curtis et al., 2018; Fancourt et al., 2021; Fancourt & Finn, 2019; Noice et al., 2014; Zarobe & Bungay, 2017). Given that 96% of arts and cultural events were canceled in the first five months of the pandemic (Americans for the Arts, 2021), understanding who engaged in home-based arts activities in the United States in this period has significant health and policy implications. Our findings should (a) reveal whether the usual predictors of arts engagement continued to affect audience profiles, or whether barriers to engagement were reduced for certain demographic groups in the United States; (b) identify people who may be at risk of being excluded from the arts during a national crisis, which is important given the link between arts engagement and wellbeing; and (c) provide direction for arts organizations (Radermecker, 2021).

## Methods

### Sample

The study was a United States extension of the United Kingdom COVID-19 Social Study run by University College London; a panel study collecting data weekly during the pandemic (https://github.com/UCL-BSH/CSSUserGuide). The COVID-19 Social Study did not recruit a random sample and is thus not representative of the United States population. However, it does contain a heterogeneous sample that was recruited using a snowballing approach with a focus on reaching diverse populations. National social, health, and arts organizations and networks shared the study invitation through their email lists and social media. We included 4,731 participants who participated between April 6 and July 23, 2020 (see Supplemental Appendix).

The study was approved by the UCL Research Ethics Committee (12467/005) and the University of Florida Institutional Review Board (IRB202000785). All participants gave informed consent.

### Measures

#### Arts Engagement

We measured arts engagement with three questions at one wave completed by participants between April 6 and July 23, 2020. These questions were taken from the United Kingdom COVID-19 Social Study, allowing for cross-country comparisons. They were part of a series of questions following a time diary approach, in which participants were asked to focus on a single day and consider how much time they spent on a range of activities. Given concerns about the cognitive burden of focusing on a “typical” day (which involves aggregating information from multiple days and averaging), we asked participants to focus just on the last weekday (Monday to Friday). Participants were told that this may be yesterday, or it may be before the weekend, as they could complete the survey on any day. We chose weekdays to remove variation in responses due to whether participants took part on weekends, when they may have more available leisure time. Participants were asked how long they had spent engaging in (a) reading for pleasure, (b) a home-based arts or crafts activity (e.g., painting, creative writing, sewing, playing music, etc.), and (c) digital arts activities (e.g., streaming a concert, virtual tour of a museum, etc.) on the last weekday. Given the low frequency of arts engagement, we created binary variables indicating whether participants had spent time on each of the activities (yes, no).

#### Predictors

We measured a range of demographic, socioeconomic, psychosocial, and health-related factors as well as adverse events and worries during lockdown. Demographic factors included age (18–29, 30–59, ≥60 years), gender (male, female), race/ethnicity (White, Black, Asian/Asian American, Mixed Race, Other [including Latino/Hispanic, Chinese/Chinese American, Middle Eastern/Middle Eastern American, Native/American Indian/Alaska Native]), marital status (never married, divorced/widowed, in a relationship/married living apart, in a relationship/married cohabiting), living arrangement (alone, not alone but no children, not alone with children), and urbanicity of home area (rural, city/town).

Socioeconomic factors were employment status (unemployed, employed), highest level of education (high school or less, some college, undergraduate degree, postgraduate/professional degree), and household income (<$75,000, ≥$75,000 household income per annum). Binary variables (yes, no) measured household overcrowding (1 person per room excluding bathrooms and kitchens), homeowner status, and keyworker status (e.g., health or social care worker, teacher or child care worker still traveling to work, transport worker, and key public services).

We included three psychosocial and two health-related measures. Social support was measured with an adapted version of the 6-item Perceived Social Support Questionnaire. Higher scores indicate more perceived social support (range 6–30; Lin et al., 2019). Large social network was indicated by the number of friends (<3, ≥3). Loneliness was measured using the three-item Revised UCLA Loneliness Scale, with an additional item asking how often respondents felt lonely. Higher scores indicate greater loneliness (range 4-12; Russell et al., 1980). Participants were asked if they had been clinically diagnosed with a mental health condition (yes, no) or a physical condition/disability (yes, no).

Finally, we considered whether six adverse events were experienced or were a source of worry during the pandemic (yes, no): (a) COVID-19 diagnosis; (b) physical/psychological abuse (physically harmed/hurt, bullied, controlled, intimidated, or psychologically hurt by someone else); (c) financial difficulties (unable to pay bills/rent/mortgage or had a major cut in household income); (d) lost work; (e) difficulties accessing food; (f) difficulties accessing medication.

### Statistical Analysis

We used logistic regression to investigate the cross-sectional associations between predictors and each type of arts engagement (reading for pleasure, arts or crafts activities, digital arts activities) in three separate models. All predictors were included in each model simultaneously.

We weighted the final analytical sample to match the characteristics of the noninstitutionalized United States population aged 18 years and above by weighting according to age, gender, race/ethnicity, and education, obtained from the United States Census Bureau (2021), using the Stata package ebalance (Hainmueller & Xu, 2013). To remove extreme variation, weights were trimmed to a maximum of the median plus 6 times the interquartile range, and then adjusted so that the total summed to the number of participants (Chowdhury et al., 2007; Potter & Zheng, 2015). For comparison, unweighted analyses are presented in Supplemental Table S1. All analyses were performed using Stata 16 (StataCorp, 2019).

## Results

Before weighting, 83% of the sample were female, 86% were of White race/ethnicity, and 47% had postgraduate/professional qualifications (Table 1). After weighting, 58% of the sample were female, 80% were of White race/ethnicity, and 18% had postgraduate/professional qualifications. Of this sample, 65% reported reading for pleasure on the last weekday, 42% did a home-based arts or crafts activity, and 20% did a digital arts activity.

**Table 1:** Sociodemographic Characteristics of the Sample and Arts Engagement on the Last Weekday

Sociodemographic characteristics	Unweighted	Weighted
Age group (years)		
18–29	15%	19%
30–59	53%	49%
60+	32%	32%
Gender		
Male	17%	42%
Female	83%	58%
Race/ethnicity		
White	86%	80%
Black	3%	10%
Asian/Asian American	2%	5%
Mixed Race	4%	3%
Other	5%	2%
Marital status		
Single, never married	20%	26%
Single, divorced or widowed	16%	17%
In a relationship/married, living apart	6%	7%
In a relationship/married, cohabiting	57%	51%
Living arrangement		
Alone	22%	20%
Not alone, no child	54%	55%
Not alone, with child	24%	25%
Urbanicity of home area		
Rural	11%	14%
City/town	89%	86%
Employment status		
Unemployed	35%	45%
Employed	65%	55%
Education		
High school or less	3%	15%
Some college	18%	37%
Undergraduate	32%	30%
Postgraduate/professional	47%	18%
High household income (US$75,000+)	55%	44%
Household overcrowded	2%	4%
Homeowner	63%	59%
Keyworker	23%	23%
Social support (range 6–30)—mean (*SD*)	23.25 (5.81)	22.04 (6.42)
Loneliness (range 4–12)—mean (*SD*)	6.96 (2.54)	7.20 (2.70)
Large social network (3+ friends)	74%	66%
Mental health problem	38%	39%
Physical health problem	47%	51%
Had COVID-19	9%	8%
Physically/psychologically abused	10%	12%
Financial difficulties	17%	21%
Lost work	9%	11%
Difficulties accessing food	3%	5%
Difficulties accessing medication	2%	3%
Worried about COVID-19	53%	50%
Worried about personal safety	21%	22%
Worried about finances	45%	47%
Worried about work	24%	23%
Worried about food access	16%	16%
Worried about medication access	10%	11%
Arts engagement on last weekday		
Read for pleasure	67%	65%
Did home-based arts or crafts activity	43%	42%
Did digital arts activity	21%	20%

*Note. N* = 4,731. Data were weighted by age, gender, race/ethnicity, and education.

### Reading for Pleasure

The odds of reading for pleasure were higher for older participants and for those with increasing levels of education (Table 2). Participants who were in a relationship but living apart also had higher odds of reading for pleasure than those who were single. In addition, participants reporting higher levels of social support had higher odds of reading for pleasure. There was no evidence that any other predictors were associated with reading for pleasure.

**Table 2:** Associations Between Predictors and Each Type of Arts Engagement

	Reading for pleasure		Arts or crafts activities		Digital arts activities	
Predictor	OR (95% CI)	*p*	OR (95% CI)	*p*	OR (95% CI)	p
Age group (vs 18–29)						
30–59	**1.45 (1.04–2.02)**	**0.029**	0.84 (0.60–1.17)	0.290	1.47 (0.94–2.30)	0.090
60+	**3.25 (2.12–4.98)**	**<0.001**	0.73 (0.49–1.11)	0.142	**2.33 (1.39–3.90)**	**0.001**
Female (vs male)	0.95 (0.75–1.19)	0.633	**1.77 (1.42–2.21)**	**<0.001**	0.78 (0.59–1.03)	0.076
Race/ethnicity (vs White)						
Black	1.48 (0.93–2.35)	0.097	**1.64 (1.06–2.53)**	**0.027**	**2.43 (1.53–3.85)**	**<0.001**
Asian/Asian American	0.91 (0.56–1.48)	0.714	1.14 (0.68–1.91)	0.614	**1.92 (1.02–3.60)**	**0.044**
Mixed Race	0.97 (0.51–1.85)	0.928	**2.15 (1.26–3.67)**	**0.005**	1.69 (0.91–3.15)	0.098
Other	1.20 (0.64–2.27)	0.570	**2.51 (1.42–4.44)**	**0.002**	**2.47 (1.28–4.78)**	**0.007**
Marital status (vs single, never married)						
Single, divorced or widowed	1.10 (0.75–1.61)	0.637	1.16 (0.79–1.68)	0.453	0.96 (0.61–1.50)	0.843
In a relationship/married, living apart	**1.73 (1.10–2.72)**	**0.018**	1.00 (0.64–1.57)	0.999	0.71 (0.39–1.30)	0.270
In a relationship/married, cohabiting	0.93 (0.66–1.29)	0.651	1.07 (0.76–1.49)	0.703	0.76 (0.50–1.15)	0.195
Living arrangement (vs alone)						
Not alone, no child	1.03 (0.73–1.46)	0.858	1.08 (0.78–1.51)	0.635	1.11 (0.74–1.68)	0.607
Not alone, with child	1.01 (0.68–1.50)	0.967	**1.47 (1.00–2.14)**	**0.047**	1.41 (0.89–2.25)	0.144
Lives in city/town (vs rural)	1.06 (0.76–1.46)	0.741	0.95 (0.69–1.31)	0.767	1.04 (0.73–1.49)	0.834
Employed (vs unemployed)	0.83 (0.64–1.07)	0.152	0.96 (0.75–1.23)	0.732	1.22 (0.90–1.65)	0.208
Education (vs high school or less)						
Some college	**1.58 (1.04–2.39)**	**0.032**	1.33 (0.90–1.96)	0.150	1.49 (0.87–2.55)	0.146
Undergraduate	**1.62 (1.07–2.46)**	**0.022**	1.00 (0.68–1.47)	0.995	1.52 (0.90–2.58)	0.116
Postgraduate/professional	**1.70 (1.11–2.60)**	**0.015**	0.94 (0.63–1.40)	0.758	1.58 (0.93–2.69)	0.091
High household income	1.22 (0.96–1.55)	0.110	0.84 (0.66–1.06)	0.142	1.18 (0.88–1.58)	0.274
Household overcrowded	0.88 (0.50–1.53)	0.641	1.02 (0.58–1.79)	0.938	0.94 (0.47–1.85)	0.852
Homeowner	1.20 (0.93–1.55)	0.163	1.10 (0.86–1.40)	0.445	1.09 (0.81–1.45)	0.566
Keyworker	0.92 (0.71–1.18)	0.499	**0.68 (0.52–0.89)**	**0.004**	**0.62 (0.45–0.85)**	**0.003**
Social support	**1.03 (1.01–1.05)**	**0.012**	**1.03 (1.00–1.05)**	**0.039**	**1.04 (1.01–1.07)**	**0.009**
Loneliness	0.97 (0.92–1.02)	0.277	0.99 (0.94–1.05)	0.750	0.99 (0.92–1.06)	0.715
Large social network	1.21 (0.94–1.56)	0.140	**1.32 (1.03–1.69)**	**0.031**	**1.43 (1.07–1.91)**	**0.016**
Mental health problem	0.94 (0.74–1.19)	0.617	0.96 (0.76–1.20)	0.692	0.95 (0.72–1.27)	0.748
Physical health problem	0.88 (0.70–1.12)	0.297	0.87 (0.69–1.09)	0.221	0.83 (0.63–1.10)	0.198
Had COVID-19	1.26 (0.86–1.83)	0.234	0.82 (0.57–1.17)	0.270	1.38 (0.89–2.13)	0.150
Physically/psychologically abused	1.24 (0.85–1.79)	0.261	**1.47 (1.04–2.09)**	**0.030**	**2.41 (1.64–3.55)**	**<0.001**
Financial difficulties	1.08 (0.79–1.49)	0.635	1.33 (0.99–1.80)	0.061	1.37 (0.98–1.92)	0.067
Lost work	1.31 (0.88–1.94)	0.183	1.37 (0.94–2.01)	0.104	1.66 (1.08–2.55)	0.021
Difficulties accessing food	1.62 (0.84–3.14)	0.153	0.91 (0.46–1.78)	0.780	1.08 (0.52–2.26)	0.837
Difficulties accessing medication	1.21 (0.52–2.77)	0.660	0.92 (0.45–1.90)	0.830	0.65 (0.29–1.45)	0.290
Worried about COVID-19	0.92 (0.73–1.16)	0.477	1.06 (0.85–1.32)	0.582	1.17 (0.89–1.52)	0.259
Worried about personal safety	1.02 (0.77–1.36)	0.888	1.12 (0.85–1.47)	0.413	1.02 (0.73–1.42)	0.900
Worried about finances	1.28 (1.00–1.65)	0.053	1.12 (0.88–1.42)	0.358	0.97 (0.72–1.30)	0.840
Worried about work	0.95 (0.72–1.26)	0.733	1.06 (0.80–1.41)	0.663	1.32 (0.95–1.84)	0.099
Worried about food access	1.25 (0.89–1.75)	0.205	0.90 (0.64–1.25)	0.514	1.06 (0.73–1.54)	0.766
Worried about medication access	0.92 (0.61–1.40)	0.713	1.39 (0.93–2.08)	0.108	1.13 (0.72–1.77)	0.603

*Note. N* = 4,731. Data were weighted by age, gender, race/ethnicity, and education. Reference categories are shown in brackets. Bold font indicates significant results at *p* < .05. OR = odds ratio; CI = confidence interval.

### Home-Based Arts or Crafts

Females had higher odds of engaging in home-based arts or crafts activities than males (Table 2). There was also evidence that participants of all races/ethnicities except Asian/Asian American were more likely to do arts or crafts activities than White participants. Those living with other adults and children were also more likely to do arts or crafts than those living alone. In addition, individuals with more social support and a larger social network had higher odds of doing arts or crafts activities, as did those who had been physically or psychologically abused. However, keyworkers had lower odds of engaging in arts or crafts activities than participants who were not keyworkers.

### Digital Arts Activities

Compared to the youngest participants, those aged 60 years and above had higher odds of engaging in digital arts activities (Table 2). There was also evidence that participants of all races/ethnicities except Mixed Race had higher odds of engaging in digital arts activities than White participants. As for arts or crafts activities, individuals with more social support and a larger social network and those who had been physically or psychologically abused had higher odds of doing digital arts activities. Participants who had lost work also had higher odds of doing digital arts activities. Keyworkers had lower odds of engaging in digital arts activities than those who were not keyworkers.

## Discussion

In this study, a range of factors were independently associated with home-based arts engagement during the first 4 months of the pandemic. Around two-thirds of participants reported reading for pleasure, over one-third did a home-based arts or crafts activity, and one-fifth did a digital arts activity on the last weekday. The strongest predictors of arts engagement were social support, social network size, age, race/ethnicity, keyworker status, and experiencing physical or psychological abuse during the pandemic. Other factors were only associated with one type of arts engagement. For example, females and those living with children were more likely to do arts or crafts and individuals in a relationship but living apart were more likely to read for pleasure. Higher levels of education were associated with increased odds of reading for pleasure, but not engaging in arts or crafts or digital arts activities. Most socioeconomic and health-related factors were not associated with arts engagement, including employment status, household income and overcrowding, loneliness, mental and physical health problems, having COVID-19, and other adverse events and worries experienced during the pandemic.

In comparison to before the pandemic, some predictors of arts engagement appear to have remained consistent. For example, higher levels of perceived social support and larger social networks were most consistently associated with arts engagement, as in prepandemic studies of home-based arts engagement (Fancourt et al., 2020) and cultural engagement more broadly (Fancourt & Baxter, 2020). This may be because individuals with more supportive relationships receive more encouragement to participate in the arts, both in the form of material resources (e.g., paying for lessons or providing tangible resources such as books) and nonmaterial resources (e.g., emotional support or awareness of online arts activities). This association may be bidirectional, as engagement could provide opportunities to connect with others and build a sense of community (Fancourt et al., 2021). We also found that females were more likely to do arts or crafts, which is in line with previous evidence before and during the pandemic (Bone et al., 2021; Mak et al., 2020, 2021; Stallings & Mauldin, 2016).

Some predictors of arts engagement in the United States may have changed during the pandemic. Although higher levels of education were associated with increased odds of reading for pleasure, education was not associated with doing arts or crafts or digital arts activities. This differs from prepandemic evidence that education is one of the strongest predictors of all forms of arts engagement (Bone et al., 2021; Stallings & Mauldin, 2016). It is possible that the increased availability of digital arts activities created new opportunities for people from varied backgrounds to become aware of the arts during the pandemic. In addition, the closure of museums, theaters, and arts venues could have predominantly impacted individuals with higher levels of education (Suarez-Fernandez et al., 2020).

Similarly, socioeconomic position is usually a strong predictor of broad arts engagement in the United States (Bone et al., 2021; Stallings & Mauldin, 2016), but we found no evidence that income was associated with home-based engagement during the pandemic, as shown in the United Kingdom during the pandemic (Mak et al., 2021). The lack of association with doing digital arts activities is particularly surprising given the link between household income and access to electronic devices and a stable internet connection. We expected that, although the rapid increase in digital arts activities during the pandemic might reach new audiences, it may also have excluded individuals without access to reliable internet and those unable to use digital technology. However, the lack of association with income could be because reading for pleasure, home-based arts or crafts, and digital arts activities are often affordable and easily accessible. In contrast, in-person cultural events can be expensive and require attendance at specific venues with associated transport costs.

Associations between race/ethnicity and arts engagement may also have changed in the pandemic. We found that reading for pleasure did not differ by race/ethnicity, but individuals who were not of White race/ethnicity were more likely to do arts or crafts and digital arts activities than people of White race/ethnicity. This may be due to the shift in location of arts activities from physical spaces rooted in white supremacy (e.g., art museums with colonial histories and racist legacies), which create a foundational barrier for Black, Indigenous, and other people of color (BIPOC groups), to digital interfaces that can be accessed from any location.

In line with findings from the United Kingdom (Mak et al., 2021), arts engagement did not differ for individuals with or without physical or mental health conditions. In contrast, prepandemic studies of community-based activities found that people with worse health and wellbeing were less likely to engage in these activities (Fancourt & Baxter, 2020; Steptoe & Fancourt, 2019). In addition to the greater accessibility and availability of home-based arts activities, individuals with existing health problems may have been more likely to use the arts to manage symptoms and cope with stress during the pandemic (Fancourt et al., 2019; Mak et al., 2021). Similarly, people who experienced physical or psychological abuse were more likely to engage in arts or crafts and digital arts activities, which might also be because these individuals were using the arts to cope and regulate their emotional responses to adverse events (Mak et al., 2021; Wright et al., 2021). Digital arts provision may also have removed physical barriers relating to access. It is particularly interesting that experiencing physical or psychological abuse and reporting more social support were both associated with increased odds of home-based arts engagement; this should be explored in future research.

Some of our findings may be a direct result of the pandemic and restrictions. Keyworkers were less likely to do arts or crafts and digital arts activities. They continued traveling to work throughout the pandemic and may have faced increased working hours, stress, and burnout (Ruiz & Gibson, 2020). Keyworkers may not have benefited from the increases in leisure time experienced by others and instead had a decreased work-life balance, making them less able to engage in arts activities. This is concerning given that this group may have particularly benefited from the arts as a coping strategy. In addition, individuals living with children were more likely to do arts or crafts activities, which might have offered opportunities for parents to engage and bond with their children, as well as preventing boredom (Choi et al., 2020). Finally, older adults were more likely to read and engage in digital arts, which could be a result of the stricter stay-at-home directives for vulnerable older adults, with additional time at home providing increased opportunities and motivations to engage.

### Strengths and Limitations

This study has several strengths, including the large sample recruited early in the pandemic. It was one of the first studies to examine arts engagement in the United States during the pandemic. A wide range of sociodemographic information was collected, allowing comparison to similar data from the United Kingdom (Mak et al., 2021). However, the COVID-19 Social Study sample was not random and may have been biased toward individuals who were more engaged in the arts as participants were recruited with the help of Americans for the Arts. While our data was weighted according to age, gender, race/ethnicity, and education distributions in the United States population (U.S. Census Bureau, 2021), we cannot rule out biases due to omitting other factors associated with survey participation in the weighting process. Given that the survey was completed online, our sample may exclude people who do not have digital access or comfort with digital interfaces. In addition, we excluded participants who did not identify as male or female to match our data to available population statistics. As the United States Census Bureau (2021) only gives people the option to report their gender as male or female, the proportion of people in the United States who do not identify as male or female is unknown, and we could not include these individuals in our sample weighting. Our weighted sample is thus not representative of all individuals in the United States. We recognize that gender is not a binary construct and changes to the measures used by the United States Census Bureau are needed. We also used an overly simple race/ethnicity variable, due to small numbers in non-White groups, which conflates experiences across diverse racial/ethnic groups. Future research must include more diverse samples and collect detailed data on race/ethnicity.

Furthermore, our measures of arts activities were limited to engagement on the last weekday. This aimed to reduce the cognitive burden of imagining a typical day and to remove differences in available leisure time between weekdays and weekends. However, it is possible that asking participants to focus on the last weekday introduced bias, as some may have only engaged in arts activities on the weekends. This could have particularly affected lower income groups, who might have worked longer hours on weekdays and had less time available for arts engagement. Yet, despite this, we did not find any evidence that engagement differed according to household income. In addition, our analysis was based on cross-sectional data, meaning causality cannot be established. It is likely that some associations were bidirectional. As data collection began after the onset of the pandemic, we were not able to compare arts engagement during the pandemic to prepandemic behavior. Future research could investigate longer-term changes in arts engagement to better understand the changing characteristics of audiences in the United States and whether differences are sustained post-pandemic.

### Implications for Policy and Research

Our findings have important implications for health professionals, arts practitioners, policy, and future research. They contradict previous evidence that digital engagement replicates, or even enlarges, existing inequalities in community-based cultural engagement (Weingartner, 2021). It is likely that the pandemic was an exceptional situation, with many barriers to accessing the arts removed, enabling engagement for people from a wide range of backgrounds. Given the numerous health benefits of arts engagement (Fancourt & Finn, 2019), the potential diversification of arts audiences during the pandemic is promising for both population-level health and wellbeing and the future of the arts sector. In addition, increasing arts engagement in historically excluded groups could counteract inequalities in the impacts of the COVID-19 pandemic (Dorn et al., 2020). For example, we found no differences in engagement for people with and without mental and physical health problems and there were higher rates of engagement in those not of White race/ethnicity. Arts practitioners and health professionals should encourage people who are not able to access community arts resources, or do not feel comfortable doing so, to do home-based arts activities, as they are likely to have similar potential health benefits (Bu et al., 2021; Curtis et al., 2018; Fancourt et al., 2021; Fancourt & Finn, 2019; Noice et al., 2014; Zarobe & Bungay, 2017). This is not just relevant during stay-at-home restrictions but also as society moves to the new normal. It is also promising that some vulnerable groups had higher levels of arts engagement during the pandemic, such as older adults and those who had experienced physical or psychological abuse. Health professionals should encourage people to use the arts to manage symptoms, cope with stress, and regulate emotional responses to adverse events (Fancourt et al., 2019; Mak et al., 2021).

Enabling keyworkers to engage in arts activities should also be a priority for policy makers and arts and health practitioners, especially during future health emergencies. This group may particularly benefit from the arts as a coping strategy (Aughterson et al., 2021; Wright et al., 2021) but were less likely to engage during the pandemic. Access to arts activities could be enhanced through workplace arts and creative programming and broader policies such as reducing working hours and ensuring that keyworkers are not under-resourced. In line with this, future research should explore how arts programming can be more responsive to community needs, as well as whether arts audiences changed further throughout the pandemic. Subsequent studies should also explore the specific conditions needed for people to continue participating following the pandemic and the conditions needed to reduce barriers to arts participation that have persisted, particularly for BIPOC groups, in the United States. This will require efforts to recruit more diverse and representative samples.

## Conclusion

Overall, our findings indicate that the social gradient in home-based arts and crafts and digital arts activities and reading for pleasure was reduced in the first 4 months of the COVID-19 pandemic in the United States. Most socioeconomic and health-related factors were not associated with arts engagement, including household income and mental and physical health problems. The factors most strongly associated with all three types of arts engagement were social support, social network size, age, race/ethnicity, keyworker status, and experiencing physical or psychological abuse during the pandemic. Our findings are generally in line with evidence from the United Kingdom, which also showed a narrowing of inequalities in arts engagement in the early months of the pandemic when individuals were restricted just to at-home engagement (Feder et al., 2022; Mak et al., 2021). Given the health benefits of arts engagement, the potential diversification of arts audiences during the pandemic is promising for both population-level health and wellbeing and the future of the arts and cultural sector.

## Supplemental Material

sj-docx-1-hpp-10.1177_15248399221119806 – Supplemental material for Who Engaged in Home-Based Arts Activities During the COVID-19 Pandemic? A Cross-Sectional Analysis of Data From 4,731 Adults in the United States Supplemental material, sj-docx-1-hpp-10.1177_15248399221119806 for Who Engaged in Home-Based Arts Activities During the COVID-19 Pandemic? A Cross-Sectional Analysis of Data From 4,731 Adults in the United States by Jessica K. Bone, Hei Wan Mak, Jill K. Sonke, Meg E. Fluharty, Jenny B. Lee, Anthony J. Kolenic, Heidi Radunovich, Randy Cohen and Daisy Fancourt in Health Promotion Practice
