# Supplementary material for: Who Engaged in Home-Based Arts Activities During the COVID-19 Pandemic? A Cross-Sectional Analysis of Data From 4,731 Adults in the United States
Source: Health Promot Pract. 2022 Sep 1;25(2):244–53. doi: 10.1177/15248399221119806 (PMC10908194; doi:10.1177/15248399221119806)
Supplement: sj-docx-1-hpp-10.1177_15248399221119806 – Supplemental material for Who Engaged in Home-Based Arts Activities During the COVID-19 Pandemic? A Cross-Sectional Analysis of Data From 4,731 Adults in the United States [file sj-docx-1-hpp-10.1177_15248399221119806.docx]

# Supplementary Materials

## Sample

The study was launched as a US extension of the UK COVID-19 Social Study run by University College London; ﻿a longitudinal study that focuses on the psychological and social experiences of adults living during the COVID-19 pandemic (<https://github.com/UCL-BSH/CSSUserGuide>). The US extension used the same measures as the UK COVID-19 Social Study and was delivered in partnership with the University of Florida Center for Arts in Medicine and Americans for the Arts, a non-profit organization whose primary focus is advancing the arts in the US (<https://www.americansforthearts.org>). In this study, we only included participants from the US extension of the COVID-19 Social Study. Data collection in the US began on 6^th^ April 2020, with participants initially completing a baseline survey, followed by weekly data collection for a maximum of 12 waves.

The COVID-19 Social Study did not include a random sample, but it does contain a heterogeneous sample recruited using a snowballing approach with a focus on reaching diverse populations. National social, health, and arts organizations and networks shared the study invitation through their email lists and social media. The study was approved by the UCL Research Ethics Committee (12467/005) and the University of Florida Institutional Review Board (IRB202000785). All participants gave informed consent. A full protocol for the primary study is available online at <https://github.com/UCL-BSH/CSSUserGuide>.

A total of 6,781 participants were recruited in the US. We selected our cross-sectional sample by including the first wave of data collected on arts engagement for each participant between 6^th^ April and 23^rd^ July 2020, leaving a sample of 5,242 participants. We then further restricted the sample to participants who were: 18 and over (n=2 excluded); male or female (so that weights could be created; n=61); and had complete data on arts engagement and sociodemographic factors (n=448). Although participants reported their gender as male, female, or other, we weighted analyses according to the statistics available from the US Census Bureau who still use a binary measure of gender (US Census Bureau, 2021), leading to the exclusion of participants reporting their gender as other. This resulted in a final analytical sample of 4,731 participants.

**Table S1.** Associations between predictors and the odds of engaging in each type of arts engagement (unweighted).

|  | **Reading for pleasure** | | **Arts or crafts activities** | | **Digital arts activities** | |
| --- | --- | --- | --- | --- | --- | --- |
|  | OR (95% CI) | p | OR (95% CI) | p | OR (95% CI) | p |
| Age group (vs 18-29) |  |  |  |  |  |  |
| 30-59 | **1.65 (1.35-2.01)** | **<0.001** | 1.07 (0.88-1.30) | 0.513 | **1.44 (1.11-1.86)** | **0.006** |
| 60+ | **3.51 (2.72-4.52)** | **<0.001** | 1.22 (0.96-1.55) | 0.100 | **2.44 (1.81-3.30)** | **<0.001** |
| Female (vs male) | 0.98 (0.82-1.17) | 0.837 | **1.98 (1.66-2.35)** | **<0.001** | 0.97 (0.80-1.19) | 0.794 |
| Race/ethnicity (vs White) |  |  |  |  |  |  |
| Black/African American | 1.30 (0.91-1.87) | 0.147 | **1.44 (1.04-2.00)** | **0.027** | **2.26 (1.60-3.20)** | **<0.001** |
| Asian/Asian American | 1.03 (0.66-1.59) | 0.901 | 1.26 (0.82-1.94) | 0.286 | 1.54 (0.93-2.53) | 0.092 |
| Mixed Race | 1.27 (0.90-1.79) | 0.177 | **1.66 (1.21-2.28)** | **0.002** | 1.15 (0.79-1.70) | 0.463 |
| Other | 0.90 (0.68-1.19) | 0.462 | **1.68 (1.28-2.22)** | **<0.001** | **1.61 (1.18-2.21)** | **0.003** |
| Marital status (vs single, never married) |  |  |  |  |  |  |
| Single, divorced or widowed | 0.85 (0.67-1.07) | 0.164 | 0.91 (0.73-1.13) | 0.410 | 0.94 (0.72-1.22) | 0.627 |
| In a relationship/married, living apart | 1.23 (0.92-1.64) | 0.165 | 0.93 (0.70-1.22) | 0.579 | 0.79 (0.56-1.12) | 0.178 |
| In a relationship/married, cohabiting | 0.82 (0.67-1.01) | 0.066 | 0.97 (0.79-1.18) | 0.760 | 0.80 (0.62-1.02) | 0.075 |
| Living arrangement (vs alone) |  |  |  |  |  |  |
| Not alone, no child | 1.11 (0.90-1.37) | 0.343 | 1.09 (0.90-1.33) | 0.380 | 1.10 (0.87-1.40) | 0.440 |
| Not alone, with child | 0.97 (0.77-1.23) | 0.825 | 1.25 (0.99-1.56) | 0.057 | 1.14 (0.87-1.50) | 0.345 |
| Lives in city/town (vs rural) | 0.92 (0.75-1.13) | 0.423 | 0.86 (0.71-1.04) | 0.118 | 0.97 (0.77-1.22) | 0.781 |
| Employed (vs unemployed) | 0.88 (0.75-1.03) | 0.103 | 0.95 (0.82-1.10) | 0.524 | 1.18 (0.99-1.41) | 0.060 |
| Education (vs high school or less) |  |  |  |  |  |  |
| Some college | 1.37 (0.93-2.01) | 0.110 | 1.14 (0.78-1.66) | 0.505 | 1.41 (0.84-2.36) | 0.192 |
| Undergrad | **1.48 (1.01-2.16)** | **0.043** | 0.97 (0.67-1.41) | 0.888 | 1.44 (0.87-2.39) | 0.161 |
| Postgrad/professional | **1.55 (1.06-2.27)** | **0.023** | 0.92 (0.64-1.34) | 0.680 | 1.47 (0.88-2.44) | 0.140 |
| High household income ($75,000+) | 1.04 (0.89-1.21) | 0.615 | **0.80 (0.70-0.92)** | **0.002** | 1.11 (0.93-1.31) | 0.241 |
| Household overcrowded | 1.03 (0.69-1.54) | 0.894 | 1.23 (0.82-1.83) | 0.312 | 1.09 (0.69-1.75) | 0.706 |
| Homeowner | **1.19 (1.02-1.38)** | **0.026** | 1.10 (0.95-1.27) | 0.200 | 1.00 (0.84-1.19) | 0.969 |
| Keyworker | **0.79 (0.68-0.92)** | **0.003** | **0.78 (0.67-0.91)** | **0.001** | **0.75 (0.62-0.91)** | **0.003** |
| Social support | **1.02 (1.01-1.04)** | **0.005** | **1.03 (1.01-1.04)** | **<0.001** | **1.02 (1.01-1.04)** | **0.004** |
| Loneliness | 0.97 (0.94-1.00) | 0.081 | 1.01 (0.98-1.04) | 0.622 | 1.02 (0.98-1.06) | 0.257 |
| Large social network (3+ friends) | **1.30 (1.11-1.52)** | **0.001** | **1.23 (1.05-1.43)** | **0.008** | **1.34 (1.11-1.62)** | **0.003** |
| Mental health problem | 0.96 (0.84-1.10) | 0.570 | 0.99 (0.87-1.13) | 0.889 | **0.81 (0.69-0.95)** | **0.011** |
| Physical health problem | 0.93 (0.81-1.06) | 0.280 | 1.00 (0.88-1.14) | 0.997 | 0.98 (0.84-1.15) | 0.834 |
| Had COVID-19 | **1.31 (1.04-1.65)** | **0.023** | 1.16 (0.94-1.44) | 0.161 | 1.10 (0.86-1.42) | 0.440 |
| Physically/psychologically abused | 1.01 (0.81-1.26) | 0.910 | **1.28 (1.04-1.58)** | **0.019** | **1.40 (1.10-1.78)** | **0.006** |
| Financial difficulties | 0.96 (0.80-1.16) | 0.706 | **1.36 (1.14-1.63)** | **0.001** | **1.28 (1.04-1.57)** | **0.020** |
| Lost work | 1.04 (0.82-1.31) | 0.739 | 1.09 (0.88-1.36) | 0.425 | **1.47 (1.15-1.88)** | **0.002** |
| Difficulties accessing food | 1.16 (0.77-1.75) | 0.480 | 1.04 (0.70-1.54) | 0.853 | 0.71 (0.43-1.15) | 0.166 |
| Difficulties accessing medication | 1.28 (0.80-2.05) | 0.294 | 1.06 (0.69-1.65) | 0.789 | 1.16 (0.69-1.94) | 0.572 |
| Worried about COVID-19 | 0.91 (0.79-1.04) | 0.162 | 1.04 (0.92-1.19) | 0.511 | 1.01 (0.86-1.18) | 0.897 |
| Worried about personal safety | 0.96 (0.81-1.13) | 0.616 | 1.15 (0.98-1.35) | 0.077 | 1.06 (0.87-1.28) | 0.566 |
| Worried about finances | **1.18 (1.02-1.37)** | **0.024** | 1.07 (0.93-1.23) | 0.329 | 1.08 (0.91-1.27) | 0.390 |
| Worried about work | 0.94 (0.80-1.11) | 0.455 | 1.04 (0.89-1.22) | 0.601 | 1.21 (1.00-1.46) | 0.051 |
| Worried about food access | 0.99 (0.82-1.20) | 0.944 | 1.08 (0.90-1.29) | 0.428 | **1.24 (1.00-1.53)** | **0.049** |
| Worried about medication access | 1.00 (0.79-1.27) | 0.998 | **1.27 (1.01-1.60)** | **0.040** | 1.06 (0.80-1.40) | 0.680 |

*Note.* N=4,731. Reference categories are shown in brackets. Bold font indicates significant results at p<0.05.
